# Argatroban in patients with acute ischemic stroke with early neurological deterioration: a cost-effectiveness analysis from the perspective of Chinese healthcare system

**DOI:** 10.3389/fphar.2025.1470373

**Published:** 2025-04-03

**Authors:** Yu Xie, Liping Hu, Ping Xu, Xianbin Guo, Junxiu Cai, Min Pan, Jie Tang, Qingtao Gong, Rong Su, Yake Lou, Yan Liu, Li Wang, Ying Yu

**Affiliations:** ^1^ Department of Neurology, Zigong Third People’s Hospital, Zigong, Sichuan, China; ^2^ Department of Cardiology, Beijing Anzhen Hospital, Beijing Institute of Heart Lung and Blood Vessel Disease, Capital Medical University, Beijing, China; ^3^ Department of Interventional Neuroradiology, Beijing Tiantan Hospital, Capital Medical University, Beijing, China

**Keywords:** argatroban, sroke, cost effectiveness, early neurological deterioration, ease

## Abstract

**Background and Objective:**

Studies have shown that argatroban improves 90-day functional outcomes in patients with acute ischemic stroke (AIS) with early neurological deterioration (END). However, its cost-effectiveness in this patient population remains unclear.

**Methods:**

A combination of a short-term decision tree and a long-term Markov model was developed to calculate the total cost and effectiveness for Chinese patients with AIS with END treated with intravenous argatroban plus standard therapy or standard therapy alone. Cost data were accessed from our institution, the China National Stroke Registry, and other public sources, while effectiveness data were obtained from the EASE trial and the China Health Statistical Yearbook 2022. The primary outcome was the incremental cost-effectiveness ratio (ICER), with secondary outcomes including total cost, total effectiveness, and incremental effectiveness. One-way sensitivity analysis, probabilistic sensitivity analysis, and scenario analysis were performed to assess certainty, uncertainty, and robustness.

**Results:**

For Chinese patients with AIS with END, treatment combining argatroban with standard therapy resulted in a lifetime cost of 138,812 Chinese Yuan (CNY), compared to 136,353 CNY for standard therapy alone. The combined treatment achieved 4.19 quality-adjusted life years (QALYs) (equivalent to 8.43 life years), while standard therapy yielded 3.78 QALYs (equivalent to 8.17 life years). This led to an ICER of 5968 CNY per QALY (9367 CNY per life year), below the willingness-to-pay threshold. One-way sensitivity analysis indicated that argatroban’s efficacy was the primary driver of the ICER, consistently remaining below the threshold. PSA showed that argatroban was highly cost-effective in over 99% of cases and dominant in 0.54% of cases. Scenario analysis confirmed the robustness of these findings across various scenarios.

**Conclusion:**

Argatroban is highly cost-effective for Chinese patients with AIS and END from the perspective of the Chinese healthcare system.

## Introduction

Stroke is the second leading cause of age-standardized deaths globally and the essential contributor to the increasing burden in number of disability-adjusted life-years (GBD 2019 Diseases and Injuries Collaborators, 2020; GBD 2021 Causes of Death Collaborators et al., 2024). In 2019, there were 12.2 million incident cases of stroke in the world, with 2.3 million of them occurring in China, according to Global Burden of Disease study (GBD 2019 Stroke Collaborators, 2021; Ma et al., 2021). Ischemic stroke constituted 62.4% of all incident strokes, encompassing a diverse range of underlying etiologies, including atherosclerotic, cardioembolic, and hematologic disorders, which may present as the initial manifestation of stroke and require distinct diagnostic and therapeutic approaches (Ma et al., 2021; Arboix and Besses, 1997). Early management in acute phase is important to lower mortality and improve functional outcome (GBD 2019 Stroke Collaborators, 2021; Powers et al., 2019). However, 13.8% of patients do not recover and even deteriorates within 48 h after acute ischemic stroke (AIS) despite of early management based on current guideline, so called early neurological deterioration (END) (Powers et al., 2019; Seners et al., 2015). END is closely associated with a higher risk of disability and death within 3 months after ischemic stroke, and effective treatment of END is critical for improving stroke outcomes (Seners et al., 2015; Siegler and Martin-Schild, 2011).

An important approach to treating END is the use of aggressive antithrombotic therapy. However, the majority of patients with END continue to experience deterioration even after antiplatelet treatment. In addition to antiplatelet resistance and hypoperfusion, the underlying reason may also be related to thrombus extension that may result from the activation of physiological coagulation cascade due to blood stasis near to the original clot. Therefore, physicians attempted to use anticoagulant treatment to inhibit thrombus extension and reduce disability and death in patients with END after AIS. Nevertheless, the studies on anticoagulant treatment for AIS over the past 20 years have not demonstrated advantages over antiplatelet treatment, because of the high risk of intracranial hemorrhage associated with existing anticoagulants, such as warfarin and heparin (International Stroke Trial Collaborative Group, 1997; The Publications Committee for the Trial of ORG 10172 in Acute Stroke Treatment TOAST Investigators, 1998; Bath et al., 2001).

Argatroban, a direct thrombin inhibitor, has the advantages of high selectivity, rapid onset and short half-life (Hosomi et al., 2007; Barreto et al., 2012). In the rat models, argatroban has been shown to effectively reduce ischemic stroke damage (Lyden et al., 2014). Adjuvant argatroban has not increased the bleeding risk in AIS patients treated with intravenous alteplase, and the rate of symptomatic intracranial hemorrhage was only 0.9%, which shows the safety of argatroban for AIS (Chen HS. et al., 2023). A recently randomized controlled trial (RCT) on patients with AIS experiencing END in China showed the significant superiority of argatroban plus standard antiplatelet treatment in improving functional outcome at 90 days compared with standard antiplatelet treatment alone (Zhang et al., 2024). However, the cost-effectiveness of adjuvant argatroban on patients with AIS with END remains unknown. The present study is conducted to explore the economic effect of argatroban for treating patients with AIS with END from the perspective of Chinese healthcare system.

## Materials and methods

This study adheres to the updated Consolidated Health Economic Evaluation Reporting Standards (CHEERS 2022) guidelines (Husereau et al., 2022).

### Ethical approval

All the data analyzed in this study were obtained from published papers or publicly accessible databases, so ethical approval from the institutional review board was not applicable.

### Participants

This study comprised two hypothetical cohorts with baseline characteristics similar to those in the EASE study (Zhang et al., 2024). Specifically, patients had a median age of 66 years, a median NIHSS score of eight at randomization, and a median NIHSS score of 4 before deterioration. Participants were older than 18 years, had AIS within 48 h, and experienced END with an increase of 2 or more points on the total NIHSS. Patients with cardiogenic cerebral embolism, a pre-stroke modified Rankin Scale (mRS) score greater than 1, intracranial hemorrhage, or treatment with tirofiban were excluded from the study.

### Intervention

Both groups in the study received standard treatment, including oral mono or dual antiplatelet therapy (aspirin and/or clopidogrel) as determined by the attending physicians, following the 2018 Chinese Stroke Association guidelines for the diagnosis and treatment of acute ischemic stroke (Chinese Society of Neurology and Chinese Stroke Society, 2018). In addition to standard treatment, patients in the intervention group received intravenous argatroban for 7 days. The dosing regimen consisted of a continuous infusion of 60 mg per day for the first 2 days, followed by 20 mg per day for the subsequent 5 days. Argatroban administration was immediately terminated if major systemic bleeding or symptomatic intracerebral hemorrhage was suspected.

### Model overview

**FIGURE 1:**
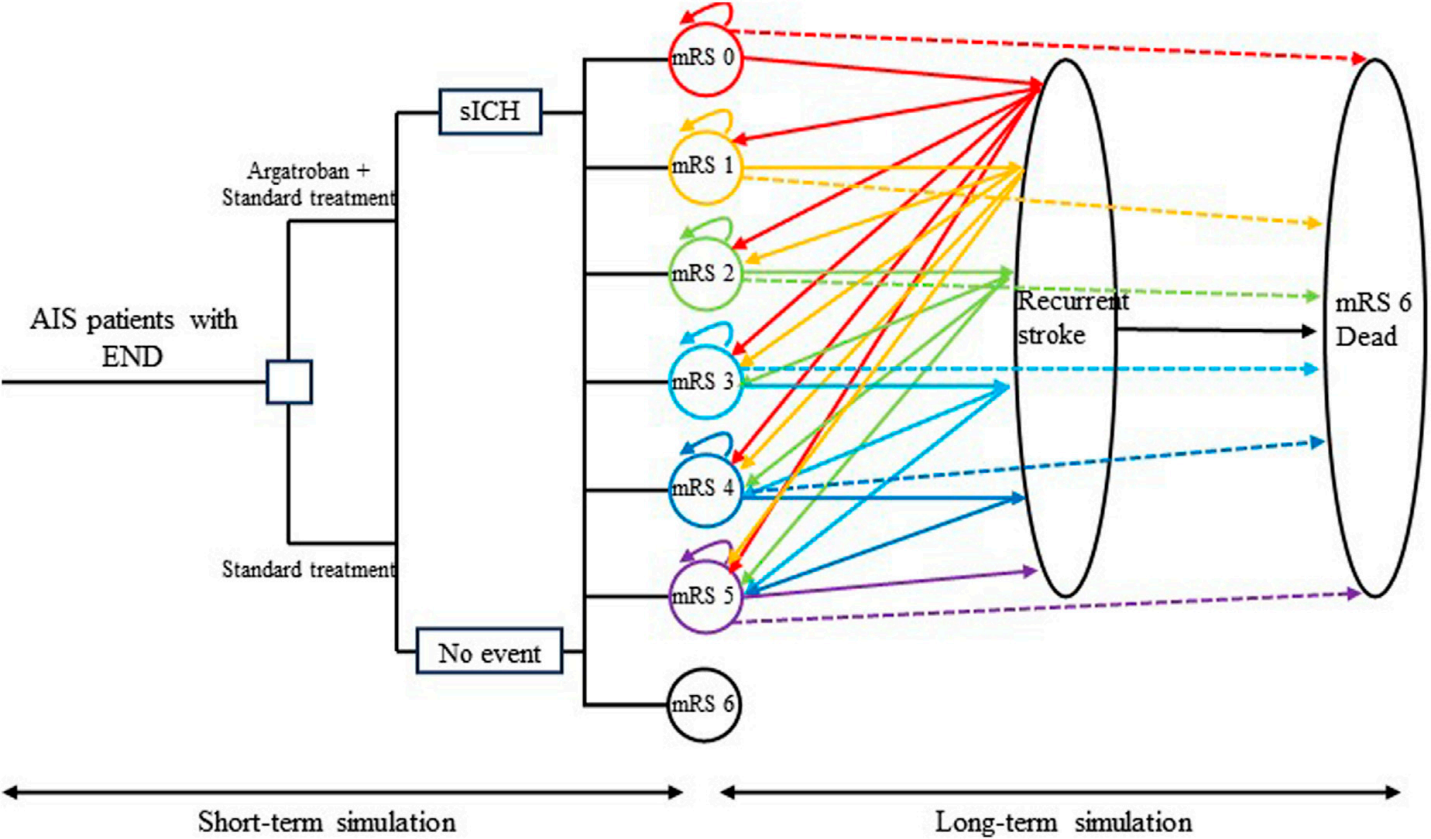
Schematic diagram of the model. The left section shows the short-term cost-effectiveness simulation, while the right section shows the long-term simulation. AIS, acute ischemic stroke; END, early neurological deterioration; sICH, symptomatic intracranial hemorrhage; mRS, modified Rankin scale.

A combination of short-term decision tree simulation and long-term Markov model simulation was developed to calculate the total cost and effectiveness for Chinese patients with AIS with END treated with intravenous argatroban plus standard therapy or standard therapy alone (Figure 1). The decision tree model estimated the total cost and effectiveness for the first 3 months, while the Markov model calculated these metrics for the subsequent period. The simulation duration was 30 years, covering the remaining life expectancy of most participants in the EASE study, with a Markov cycle length of 3 months, resulting in 119 cycles in the Markov model, in addition to the first 3 months covered by the decision tree. The total cost was calculated by summing the costs from the decision tree and the Markov model, and the effectiveness was calculated using the same method. Considering that the efficacy of argatroban for AIS with END has only been validated in Chinese patients, this study explored the cost-effectiveness of adding argatroban for Chinese patients with AIS with END within the Chinese healthcare setting. Only direct medical costs were included in the analysis, indirect costs and direct non-medical costs were not considered.

Several assumptions were made to better reflect the natural progression of the disease. Each patient could experience only one clinical event per cycle. If a patient had a recurrent stroke during a Markov cycle, they were not able to transition to a lower disability state in the following cycle, unless they remained stable for an entire cycle. Patients who survived a recurrent stroke were assumed to be equally distributed among disability categories of the same or greater severity. Non-stroke mortality was considered for both groups, with rates based on the mRS classification rather than treatment type. Finally, transition probabilities after discharge were influenced by the mRS score and the occurrence of recurrent strokes, regardless of whether argatroban was administered during the initial hospitalization.

In the decision tree model, eligible patients were randomly assigned to receive either intravenous argatroban plus standard therapy or standard therapy alone. The distribution of the mRS scores 3 months after stroke differed between the groups due to the varying treatment regimens. These data were directly obtained from the EASE study, a RCT investigating the efficacy of argatroban in Chinese patients with AIS with END. Subsequently, the mRS distribution at 3 months post-stroke was used to populate the initial mRS states in the Markov model.

In the Markov model, there were seven states: “mRS 0,” “mRS 1,” “mRS 2,” “mRS 3,” “mRS 4,” “mRS 5,” and “mRS 6 (Dead),” representing distinct levels of disability. Patients in these states could experience “recurrent stroke” or “death.” Those experiencing a recurrent stroke would not transition to a lower mRS classification in the subsequent 3 months. Patients who experienced death would transition to the “mRS 6 (Dead)” state and terminate their participation in the Markov cycle. It is worth noting that patients in the Markov states could die from either recurrent stroke or non-stroke-related causes. Given the higher non-stroke-related mortality in the stroke population compared to the general population, a hazard ratio was employed to adjust the non-stroke-related mortality of stroke patients (Figure 1).

Symptomatic intracranial hemorrhage (sICH) is a serious adverse event in the treatment of AIS, especially for patients receiving intravenous thrombolysis. Although the EASE study found no statistically significant difference in the incidence of sICH between treatment groups, sICH was included in our analysis, accounting for additional costs and disutility for those experiencing this complication.

### Transition probabilities

As mentioned above, the initial mRS distribution in the Markov model was directly obtained from the EASE study. It was assumed that the prognosis of stroke patients who survived AIS with END was determined by this initial mRS classification distribution in the Markov model, regardless of whether argatroban was administered during the acute phase. This means that patients with the same mRS classification in both groups had the same transition probabilities to other Markov states. The annual incidence of recurrent stroke in the Markov model was derived from Chinese stroke patients, which was 0.112, and the post-recurrent stroke mortality rate was 0.21 (Xu et al., 2007; Wang et al., 2024). To fit the Markov model, the incidence of recurrent stroke was converted into a transition probability using the formula “p = 1 - exp(-r)” and “r = - ln (1 - R)/4”, where p represents the transition probability, r represents the 3-month event rate, and R represents the annual incidence of the event. Non-stroke-related mortality was calculated by multiplying the background mortality of the same age population in China by the hazard ratio (HR) of the stroke population (Wang et al., 2024; Wang et al., 2023). Background mortality data were obtained from the China Health Statistical Yearbook 2022, published by the Chinese government (Wu, 2022). Furthermore, patients who survived a recurrent stroke were assumed to be evenly distributed across the same or higher mRS classifications (Table 1).

**TABLE 1:** Key input parameters of the model.

Parameters	Value	Range	Distribution	Source
mRS distribution at month 3 in control				
mRS 0	0.116	0.080–0.152	Dirichlet	Zhang et al. (2024)
mRS 1	0.211	0.165–0.257		
mRS 2	0.175	0.132–0.218		
mRS 3	0.231	0.184–0.278		
mRS 4	0.112	0.077–0.148		
mRS 5	0.092	0.060–0.125		
mRS 6	0.063	0.035–0.090		
Risk ratio of mRS distribution in the argatroban				
mRS 0	1.63	1.10–2.40	Lognormal	Zhang et al. (2024)
mRS 1	0.99	0.72–1.34		
mRS 2	0.92	0.64–1.32		
mRS 3	1.07	0.81–1.43		
mRS 4	0.90	0.56–1.43		
mRS 5	0.58	0.32–1.05		
mRS 6	0.64	0.32–1.30		
Probability of sICH in argatroban	0.009	0–0.020	β	Zhang et al. (2024)
Probability of sICH in control	0.007	0–0.018	β	Zhang et al. (2024)
Recurrent stroke in China^a^	0.112	0.096–0.128	β	Wang et al. (2024)
Death after recurrent stroke in China	0.21	0.189–0.232	β	Wang et al. (2024)
Death hazard ratios				
mRS 0	1	1–1.2	Lognormal	Wang et al. (2024), Wang et al. (2023)
mRS 1	1	1–1.2		
mRS 2	1.11	1–1.3		
mRS 3	1.27	1.02–1.52		
mRS 4	1.71	1.37–2.05		
mRS 5	2.37	1.9–2.84		
Background mortality in China				
66–69	0.01266	—		Wu et al. (2022)
70–74	0.02159			
75–79	0.03731			
80–84	0.0634			
85-	0.1512			
Utilities				
mRS 0	0.95	0.94–0.96	β	Wang et al. (2014)
mRS 1	0.89	0.87–0.96		
mRS 2	0.67	0.54–0.83		
mRS 3	0.44	0.29–0.60		
mRS 4	0.16	0.09–0.23		
mRS 5	0.1	0–0.21		
mRS 6	0	—		
Stroke recurrence in China	0.42	0.11–0.71	β	
Disutility of sICH	0.38	0.30–0.46	β	Xu et al. (2007), Wang et al. (2024)
Discount rate	0.05	0–0.08	—	Liu et al. 2020
Cost in China				
Argatroban (CNY per mg)	1.61	1.58–7.05	γ	Yaozh (2025)
Acute stroke (mRS 0–1)	12,472	7,204–15,704		Xu et al. (2007), Wang et al. (2024)
Acute stroke (mRS 2–5)	16,490	9,063–21,624		
Acute stroke (death)	14,133	6,640–18,679		
sICH	3,012	654–6,285		
Annual posthospitalization (mRS 0–1)	8,867	2,655–11,311		
Annual posthospitalization (mRS 2–5)	13,492	3,393–16,968		
Recurrent stroke	18,380	13,785–22,976		
IV infusion within 1 h (CNY)	15.6	5–30		Xu et al. (2007), Wang et al. (2024)
IV infusion, additional 1 h (CNY)	1	0.5–2		

mRS, modified Rankin Scale; sICH, symptomatic intracranial hemorrhage; IV, intravenous; CNY, Chinese Yuan.

^a^ To convert an annual incidence of 0.112 to a 3-month transition probability, use r = - ln (1 - R)/4 and then p = 1 - exp(-r), where R is the annual incidence.

### Costs

All costs in this study were unified to 2023 values in Chinese Yuan (CNY). For costs incurred before 2023, they were adjusted to 2023 values using the healthcare consumer price index in China over the past few years, which were 1.027, 1.038, 1.06, 1.043, 1.024, 1.018, 1.004, 1.006, and 1.011, from 2015 to 2023 (National Bureau of Statistics, 2025). Future costs were discounted at a rate of 0.05, according to the China Guidelines for Pharmacoeconomic Evaluations (Liu, 2020).

The cost of argatroban in China varied among different manufacturers, ranging from 1.58 to 25.5 CNY per mg. As the EASE study did not disclose a specific manufacturer of argatroban, we adopted the median cost of argatroban in China (1.614 CNY per mg) for the base case analysis. This median cost was obtained from the collective purchasing prices listed by the Chinese government (Yaozh, 2025). The total dosage of argatroban for each patient in the intervention group was 220 mg, amounting to 355 CNY per patient. Additionally, the cost of intravenous infusion was included in our analysis (Table 1).

Stroke-related costs encompassed several aspects, including the cost of treatment in the acute phase, usual care after discharge, recurrent stroke, and serious adverse events (SAEs). In clinical practice, higher mRS classifications correlate with higher costs, both in the acute phase and during usual post-stroke care (Table 1).

To more accurately reflect the overall cost of stroke treatment during the acute phase and usual care after discharge across China, the cost data were derived from the China National Stroke Registry (CNSR), the largest stroke registry study in the country. This registry prospectively enrolled 21,902 consecutive patients diagnosed with acute cerebrovascular events from 132 hospitals, covering all 27 provinces and four municipalities in China. Clinical and functional data were collected, providing a comprehensive overview of the costs associated with stroke treatment nationwide (Wang et al., 2024; Wang et al., 2023). These costs were adjusted using the domestic healthcare CPI to reflect 2023 values. The cost of recurrent stroke was obtained from a published paper reporting the cost of recurrent stroke in a Chinese healthcare institution (Wang et al., 2024; Chen J. et al., 2023). Additionally, the cost of sICH in China was obtained from the Thrombolysis Implementation and Monitor of Acute Ischemic Stroke in China (TIMS-China) database (Table 1) (Liao et al., 2013).

### Utility

Utility was employed to reflect the quality of life in stroke patients, and disutility was used to reflect the loss of the quality of life in stroke patients. In our analysis, the utility of different mRS was derived from a published paper investigating the quality of life in Chinese stroke patients, where the utility was score was calculated using the population-based preference weights for each dimension of EQ-5D, and the Chinese preference weights was used (Table 1) (Wang et al., 2014; Liu et al., 2014).

The effectiveness, united in quality-adjusted life year (QALY), was calculated by multiplying the number of life years with the utility of mRS, to comprehensively reflect the life years and quality of life. As for future effectiveness, the same discount rate with that of cost was accounted into the analysis.

Disutilities were used for recurrent stroke and sICH, as these events would cause loss of quality of life in stroke patients (Table 1).

### Outcomes

The primary outcome of the study was the incremental cost-effectiveness ratio (ICER), expressed in CNY per QALY, representing the incremental cost per incremental QALY gained. Argatroban was considered highly cost-effective if the ICER was below the willingness-to-pay threshold of one time the *per capita* gross domestic product (GDP) in China for 2023, which was 89,358 CNY. It was deemed cost-effective if the ICER fell between one and three times the *per capita* GDP, and not cost-effective if the ICER exceeded three times the *per capita* GDP, following the recommendations of the China Guidelines for Pharmacoeconomic Evaluations (Liu, 2020). Secondary outcomes included total cost, total effectiveness, incremental cost, incremental effectiveness, and total life years in both groups.

### Sensitivity analysis

Sensitivity analysis was conducted to assess the robustness of the study findings. One-way sensitivity analysis involved calculating the ICER while varying input parameters, with results depicted using a Tornado diagram. Probabilistic sensitivity analysis (PSA) was carried out through 10,000 Monte Carlo simulations using probabilistic sampling. In the PSA, costs followed a gamma distribution, transition probabilities and utilities followed a beta distribution, modified Rankin Scale (mRS) scores followed a Dirichlet distribution, and relative risks (RR) followed a lognormal distribution. PSA results were presented using a cost-effectiveness plane and a cost-effectiveness acceptability curve. Additionally, scenario analysis explored various conditions, including scenarios with the highest cost of argatroban, different genders and various ages.

## Results

In the base case analysis for a Chinese patient aged 66 with AIS with END, the lifetime cost was 138,812 CNY when treated with argatroban plus standard therapy, compared to 136,353 CNY with standard therapy alone. The corresponding effectiveness was 4.19 QALYs for the combination treatment and 3.78 QALYs for standard therapy, resulting in an ICER of 5968 CNY per QALY, which is lower than the *per capita* GDP of China in 2023. Without considering quality of life, the effectiveness was 8.43 life years and 8.17 life years, respectively, resulting in an ICER of 9,367 CNY per life year (Table 2).

**TABLE 2:** Results of base case and scenario analysis.

	Total cost (CNY)	Total effectiveness (QALY)	Total effectiveness (LY)	Incremental cost (CNY)	Incremental effectiveness (QALY)	Incremental effectiveness (LY)	ICER (CNY per QALY)
Base case							
Control	136,353	3.78	8.17				
Argatroban	138,812	4.19	8.43	2,459	0.41	0.26	5,968
Highest cost of argatroban in China							
Control	136,353	3.78	8.17				
Argatroban	144,067	4.19	8.43	7,714	0.41	0.26	18,724
Male patients							
Control	129,089	3.62	7.69				
Argatroban	131,555	4.02	7.95	2,466	0.40	0.26	6,207
Female patients							
Control	143,820	3.94	8.65				
Argatroban	146,241	4.37	8.92	2,421	0.43	0.26	5,683
Starting age of 60							
Control	153,923	4.12	9.30				
Argatroban	156,455	4.56	9.58	2,533	0.44	0.28	5,724
Starting age of 70							
Control	122,226	3.47	7.25				
Argatroban	124,623	3.85	7.50	2,397	0.38	0.25	6,265

CNY, chinese yuan; QALY, quality-adjusted life year; LY, life year; ICER, incremental cost-effectiveness ratio.

### Sensitivity analysis

**FIGURE 2:**
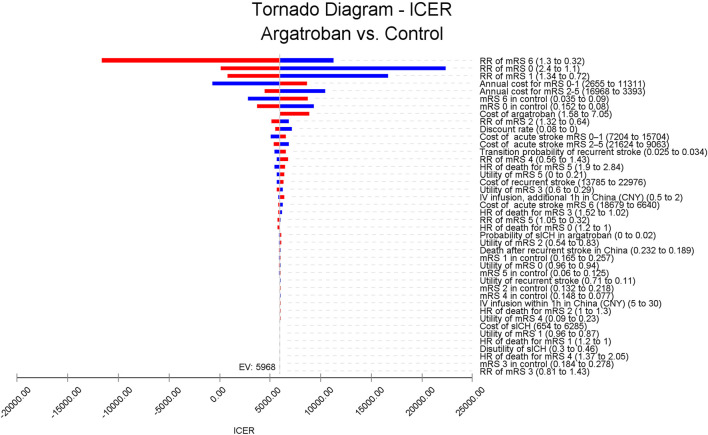
Tornado diagram of the ICER for argatroban compared to standard treatment in patients with acute ischemic stroke and early neurological deterioration. The RR of mRS of 6, 0, and 1 have the largest impact on ICER fluctuation, while the cost of argatroban has minimal impact on the ICER. ICER, incremental cost-effectiveness ratio; RR, risk ratio; mRS, modified Rankin Scale; HR, hazards ratio; IV, intravenous; CNY, Chinese Yuan; sICH, symptomatic intracranial hemorrhage.

As shown in Figure 2, the RR of mRS of 6, 0, and 1 had the largest impact on ICER fluctuations. However, these factors did not cause the ICER to exceed the WTP threshold of 89,358 CNY per QALY. Conversely, RR of mRS 6 and the annual cost for mRS 0–1 could result in an ICER below 0. Specifically, if the RR of mRS 6 was 1.3, argatroban might lead to less effectiveness compared to standard treatment. On the other hand, if the annual cost for mRS 0–1 decreased to 2,655 CNY, argatroban would be dominant, meaning argatroban plus standard treatment would incur less cost and result in greater effectiveness. For other key parameters, increasing the cost of argatroban raised the ICER, with a value of 8,871 CNY per QALY when the cost of argatroban was 7.05 CNY per mg. The recurrence rate also impacted the ICER, with a higher recurrence rate leading to a higher ICER, but still remaining below the WTP threshold. Other parameters, such as the utilities for recurrent stroke and mRS states, had minimal impact on the ICER (see Supplementary Table S1).

**FIGURE 3:**
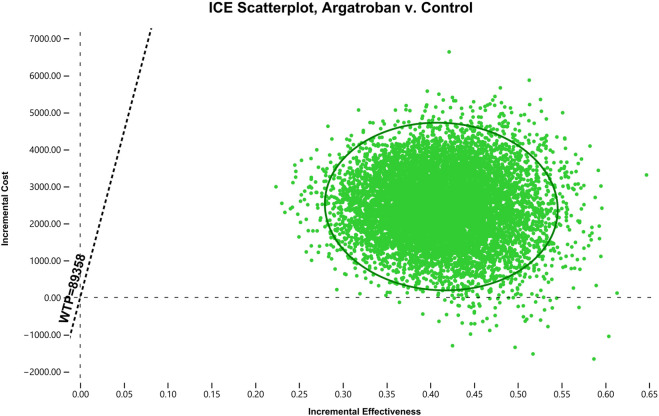
Scatter plot of the ICER comparing argatroban to standard treatment. The horizontal axis represents the incremental effectiveness (QALY), the vertical axis represents the incremental cost (CNY), and the dashed line represents the WTP threshold. It can be observed that almost all of the probability-sensitive sampling points fall below the WTP line. ICER, incremental cost-effectiveness ratio; ICE, incremental cost-effectiveness; WTP, willingness-to-pay; QALY, quality-adjusted life year; CNY, Chinese Yuan.

In the PSA, at the current WTP threshold of 89,358 CNY per QALY, argatroban was highly cost-effective in over 99% of cases and dominant in 0.54% of cases (Figure 3). Additionally, the cost-effectiveness acceptability curve indicates that at a WTP threshold of 6,000 CNY per QALY, argatroban had similar acceptability to standard treatment. Argatroban was more acceptable than standard treatment at the current WTP threshold of 89,358 CNY per QALY (see Supplementary Table S1).

In the scenario analysis, the cost of argatroban, gender, and starting age of simulation impacted cost and effectiveness. However, the corresponding ICER ranged between 5,683 and 18,724 CNY per QALY (Table 2).

## Discussion

The first RCT on adjuvant argatroban for patients with AIS with END showed that argatroban plus antiplatelet therapy can improve functional outcomes (mRS of 0–3: 80.5% vs. 73.3%; RR: 1.10, 95% CI: 1.01–1.20; p = 0.04) at 90 days compared to antiplatelet therapy alone, although it did not demonstrate superiority in reducing mortality (4% vs. 6.3%) (Zhang et al., 2024). However, there is a lack of robust evidence for the cost-effectiveness of argatroban for patients with AIS with END. To our knowledge, this economic evaluation is the first study to explore the cost-effectiveness of adjuvant argatroban in AIS patients with END. We found that adjuvant argatroban was highly cost-effective (an ICER of 5,968 CNY per QALY) in patients with AIS with END in China. Furthermore, this conclusion was confirmed through the one-way sensitivity analysis, PSA, and scenario analysis, which suggests that adding argatroban to the standard treatment for AIS patients with END is reasonable in China.

Argatroban is a selective, small-molecule direct thrombin inhibitor that has a lower risk of bleeding than other anticoagulants when used to prevent thrombus propagation after stroke onset (Barreto et al., 2012; Jeske et al., 2010). A previous small-sample size study showed that the argatroban plus tissue-type plasminogen activator (tPA) is safe in patients with AIS due to proximal intracranial occlusion and may contribute to vascular recanalization than tPA alone (Barreto et al., 2012). However, a recent RCT (the ARAIS study) investigating the effect of combining argatroban and intravenous alteplase on neurologic function in patients with AIS found that argatroban plus alteplase did not lead to higher likelihood of excellent functional outcome (mRS 0–1) at 90 days compared to alteplase alone (Chen HS. et al., 2023). The results about the effect of argatroban on neurologic function was opposite in the EASE study and the ARAIS study, possibly because of the different patients included in the two studies (Chen HS. et al., 2023; Zhang et al., 2024). The ARAIS study included patients with AIS within 4.5 h who need intravenous alteplase, while the EASE study included patients with AIS with END within 48 h. One important mechanism of END is the activation of coagulation cascade caused by the blood stasis around the original thrombus (Seners et al., 2017). Therefore, argatroban, as a safe anticoagulant, can salvage ischemic brain tissue and improve functional outcomes in patients with AIS and END by preventing the formation of new clots and the extension of existing ones (Xu et al., 2023).

While clinical trials such as the EASE study have demonstrated a low rate of sICH with argatroban, concerns remain regarding its long-term safety and bleeding risk in real-world practice. The cumulative risk of bleeding, particularly when argatroban is used in combination with other anticoagulants or in prolonged treatment regimens, warrants further investigation. Recent meta-analyses indicated that argatroban, when combined with antiplatelet drugs or intravenous thrombolytic drugs, did not significantly increase the risk of major hemorrhagic events, yet data on extended use beyond the acute phase remain limited (Cheng et al., 2024; Chlorogiannis et al., 2024). However, a trial reported that adjunctive argatroban in AIS patients receiving thrombolysis was associated with increased mortality at 90 days, suggesting that its risk-benefit profile may vary across different patient populations (Adeoye et al., 2024). These findings highlight the need for further studies to assess the long-term safety and optimal treatment duration of argatroban, particularly in combination therapies.

The primary reason why argatroban is highly cost-effective for Chinese patients with AIS with END is that argatroban improves their functional outcome and reduces mortality. The results of the one-way sensitivity analysis showed that the three most significant factors impacting the ICER were the efficacy of argatroban for AIS with END, specifically the RR of mRS of 6, 0, and 1. After a 30-year simulation, the incremental effectiveness of argatroban was 0.41 QALY and 0.26 life year, respectively, suggesting that adjuvant argatroban can improve both the life quality and life expectancy in Chinese patients with AIS with END. In addition to the efficacy of argatroban, the annual cost was another crucial factor impacting the ICER in the one-way sensitivity analysis, possibly because many patients presented with varying degrees of disability after AIS with END. The cost of care for patients with disabilities after a stroke is high and positively correlated with the mRS grading (Pan et al., 2014). Argatroban can reduce the annual cost after a stroke by improving mRS scores in Chinese patients with AIS with END.

In the present study, we found that the impact of the cost of argatroban on our results was small. One important reason may be that the price of argatroban significantly reduced after implementing China’s national centralized drug procurement (NCDP) policy (Zhu et al., 2023). The NCDP was initiated by the Chinese central government with the objectives of reducing drug prices and enhancing access of affordable medications. The drugs included in the NCDP list can be used by the vast majority of patients in China. We used the median price of NCDP in China in the base case analysis owing to the different NCDP price of argatroban from different manufacturers and the absence of disclosure on which manufacturer provided argatroban in the EASE study. Moreover, we used the first quartile and third quartile prices of NCDP as the lower and upper costs of argatroban in the one-way sensitivity analysis, and the highest price of the NCDP as the cost in the scenario analysis. The results indicated that the conclusion-which adjuvant argatroban was highly cost-effective in improving functional outcomes for AIS with END in China-was consistent across different sensitivity analyses.

It is important to note that our conclusion regarding the cost-effectiveness of argatroban was derived from the perspective of the entire Chinese healthcare system. However, regional disparities in stroke treatment costs and healthcare resource distribution were not explicitly accounted for. These disparities could introduce several challenges, such as the limited availability of argatroban in less developed areas, variations in care costs between regions, and differences in the quality of care provided at secondary versus tertiary hospitals. As a result, our findings may not be fully generalizable to all regions of China, and local healthcare resource availability should be considered when applying these conclusions. Furthermore, our analysis adopted a willingness-to-pay (WTP) threshold of 268,074 CNY per QALY, equivalent to three times China’s *per capita* GDP in 2023, as recommended by the China Guidelines for Pharmacoeconomic Evaluations. However, there is no officially established WTP threshold in China, making the appropriateness of this value uncertain. Since WTP thresholds vary significantly across countries, this variation directly influences the acceptability of argatroban. Future research, particularly studies incorporating real-world data, could help refine the estimation of an appropriate WTP threshold and its implications for the cost-effectiveness of treatments like argatroban.

Recent evidence suggests that argatroban may be beneficial for minor ischemic stroke and lacunar infarcts by reducing END and improving functional outcomes (Jin et al., 2024). Notably, lacunar strokes are associated with low acute mortality but increasing rehabilitation needs over time due to recurrent vascular events and cognitive decline (Arboix et al., 2010). Given the distinct coagulation profiles in small-vessel disease—potentially driven by endothelial dysfunction rather than thrombin-driven thrombosis—the cost-effectiveness of argatroban in this subgroup may differ (Arboix et al., 2010). A recent meta-analysis suggested that argatroban combined with antiplatelet therapy significantly improves functional outcomes in non-cardioembolic strokes (Cheng et al., 2024), yet lacunar-specific data are lacking. Similarly, cost-effectiveness analyses in minor stroke remain scarce despite promising clinical efficacy. Future studies should target the cost effectiveness of argatroban for lacunar infarcts or minor ischemic stroke populations, particularly in patients at risk of END.

This study has several limitations. First, it is based on a mathematical model that incorporates various assumptions to simulate the natural progression of the disease, rather than being a real-world study. As such, the findings require further validation through additional research. Second, although the short-term efficacy and safety of argatroban in these patients have been validated (Cheng et al., 2024; Jin et al., 2024; Yan et al., 2025), no studies have yet investigated its long-term effects on stroke recurrence. Additionally, the mRS classifications with a limited follow-up period in the EASE trial may not fully reflect long-term outcomes in the simulation. Third, this cost-effectiveness analysis relies on data from a single RCT with restrictive recruitment and a relatively short follow-up period. Results from a meta-analysis incorporating data from more RCTs could provide more robust evidence. Finally, since our study was conducted in a Chinese population, the findings may not be fully generalizable to populations in other countries. Despite these limitations, this study is the first cost-effectiveness analysis of argatroban in improving functional outcomes for AIS patients with END, demonstrating its high cost-effectiveness in China. It provides novel insights into the treatment of this population.

## Conclusion

Incorporating argatroban into the standard treatment for Chinese patients with AIS with END leads to increased costs and improved effectiveness. Given the current WTP threshold, argatroban is deemed highly cost-effective within the Chinese healthcare system. Further real-world studies are required to confirm this finding.

## Data Availability

The original contributions presented in the study are included in the article/Supplementary Material, further inquiries can be directed to the corresponding authors.
