# Supplementary material for: Argatroban in patients with acute ischemic stroke with early neurological deterioration: a cost-effectiveness analysis from the perspective of Chinese healthcare system
Source: Front Pharmacol. 2025 Apr 3;16:1470373. doi: 10.3389/fphar.2025.1470373 (PMC12003108; doi:10.3389/fphar.2025.1470373)
Supplement: Supplementary file 1 [file Supplementaryfile1.docx]

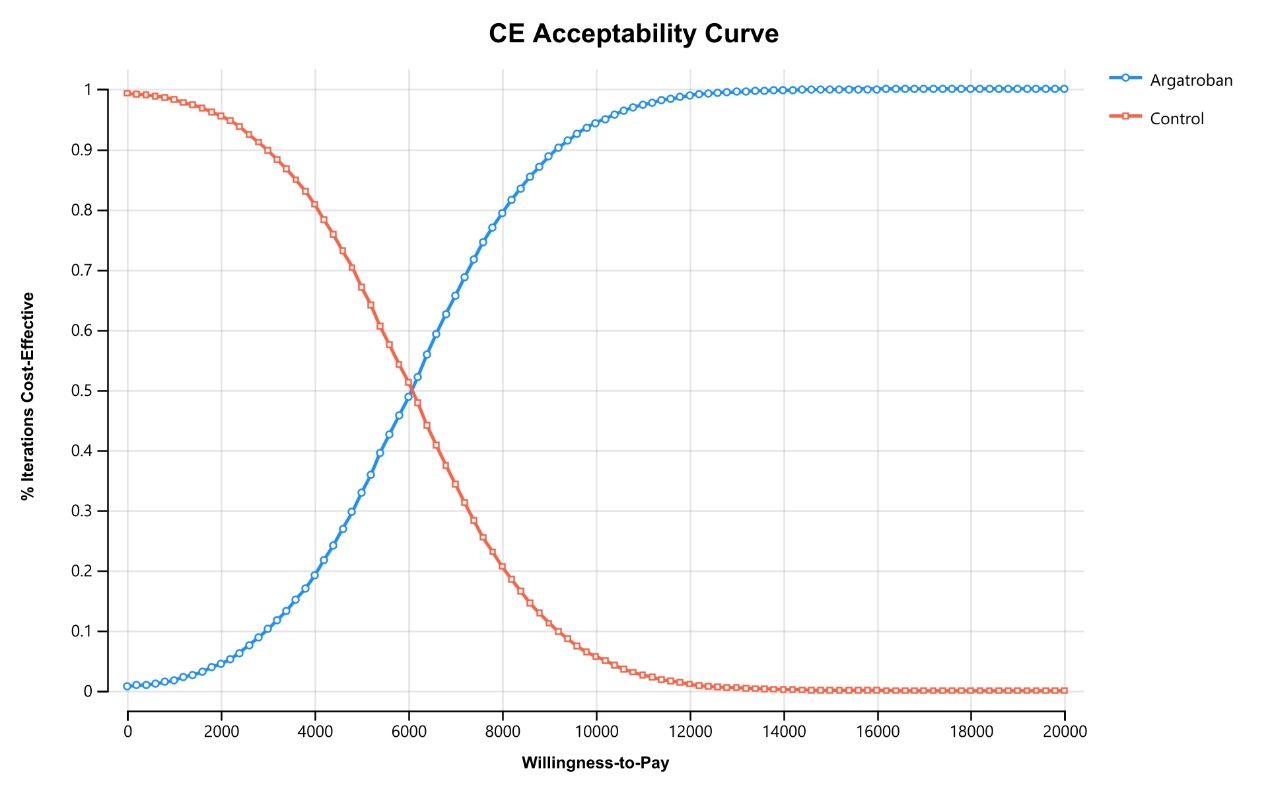


**sFigure 1. Cost-effectiveness acceptability curve.** Argatroban has higher acceptability than the standard treatment at a willingness-to-pay threshold of 6,000 CNY per QALY, which is much lower than the current threshold of 89,358 CNY per QALY.

sTable 1. The impact of key input parameters on the ICER

| Variable description | Variable Low | Variable Base | Variable High | Impact | Low | High |
| --- | --- | --- | --- | --- | --- | --- |
| Cost of argatroban | 1.58 | 1.614 | 7.05 | Increase | 5950.2701 | 8871.33314 |
| Recurrence rate | 0.025 | 0.02926 | 0.034 | Increase | 5440.15423 | 6557.52519 |
| Utility of mRS 5 | 0 | 0.1 | 0.21 | Increase | 5631.93323 | 6388.27794 |
| Utility of mRS 3 | 0.29 | 0.44 | 0.6 | Decrease | 5669.12276 | 6279.22125 |
| Utility of mRS 2 | 0.54 | 0.67 | 0.83 | Increase | 5881.21915 | 6079.37523 |
| Utility of mRS 0 | 0.94 | 0.95 | 0.96 | Decrease | 5907.99151 | 6030.11093 |
| Utility of recurrent stroke | 0.11 | 0.42 | 0.71 | Decrease | 5917.03575 | 6024.35807 |
| Utility of mRS 4 | 0.09 | 0.16 | 0.23 | Increase | 5946.6076 | 5990.40634 |
| Utility of mRS 1 | 0.87 | 0.89 | 0.96 | Decrease | 5952.36187 | 5973.03248 |

ICER, incremental cost-effectiveness ratio; mRS, modified Rankin Scale
